# Endoscopic Internalization by Cutting the Endoscopic Transpapillary Nasogallbladder Drainage Tube in Management of Acute Cholecystitis: A Retrospective Multicenter Cohort Study

**DOI:** 10.3390/jcm11247415

**Published:** 2022-12-14

**Authors:** Akinori Maruta, Takuji Iwashita, Kensaku Yoshida, Keisuke Iwata, Shogo Shimizu, Masahito Shimizu

**Affiliations:** 1 Department of Gastroenterology, Gifu Prefectural General Medical Center, Gifu 500-8717, Japan; 2 First Department of Internal Medicine, Gifu University Hospital, Gifu 501-1194, Japan; 3 Department of Gastroenterology, Gifu Municipal Hospital, Gifu 500-8513, Japan

**Keywords:** palliative, internalization, endoscopic retrograde cholangiopancreatography, endoscopic ultrasound, gallbladder drainage

## Abstract

Background: Both endoscopic nasogallbladder drainage (ENGBD) and endoscopic gallbladder stenting (EGBS) are effective management for acute cholecystitis, although ENGBD can cause discomfort due to its nature of external drainage. Converting ENGBD to EGBS after improvement of cholecystitis might be one treatment strategy. The drainage tube of ENGBD could be endoscopically cut inside the stomach to convert to internal drainage without additional endoscopic retrograde cholangiography (ERCP). Aims: To evaluate the feasibility, efficacy and safety of endoscopic internalization by cutting an ENGBD tube for acute cholecystitis. Methods: Twenty-one patients who underwent endoscopic internalization by cutting the ENGBD tube were enrolled in this study. We initially placed an ENGBD tube for gallbladder lavage and continuous drainage. After improvement of cholecystitis, the tube was cut in the stomach by esophagogastroduodenoscopy (EGD) and placed as EGBS until surgery. Results: The technical success rate of this procedure was 90.5% (19/21), and the clinical success rate was 100% (19/19). The median procedural time was 5 min (range: 2–14 min). Procedural-related adverse events (AEs) were observed in two patients where the tip of the ENGBD tube migrated into the common bile duct from the gallbladder during the procedure in both. During the waiting period for elective surgery, no AEs were identified, except for stent migration without symptoms in one patient (4.7%). Conclusion: Endoscopic internalization by cutting the ENGBD tube after improvement of cholecystitis could be an effective and safe treatment option for preventing recurrent cholecystitis in the waiting period until cholecystectomy.

## 1. Introduction

Percutaneous transhepatic gallbladder drainage (PTGBD) has traditionally been performed to drain the gallbladder and control infection [1,2,3]. However, PTGBD is contraindicated in patients with massive ascites, coagulopathy, or percutaneous inaccessible anatomical location of the gallbladder [4]. Furthermore, the external drainage tube can cause discomfort or pain at the puncture point and can potentially deteriorate the quality of life (QOL) due to limitations in activities of daily living. Endoscopic transpapillary gallbladder drainage (ETGBD) has emerged as an alternative therapy for patients with cholecystitis for whom PTGBD is contraindicated, and there have been multiple reports on the usefulness and safety of ETGBD in patients with acute cholecystitis [5,6,7,8,9,10,11,12,13,14,15]. ETGBD consists of two types of derange methods: endoscopic nasogallbladder drainage (ENGBD) as an external drainage method and endoscopic gallbladder stenting (EGBS) as an internal drainage method. ENGBD enables monitoring the drainage state, lavage of the gallbladder, and sampling of drainage fluid for bacteriological culture or pathological analysis [16]. However, ENGBD can cause significant discomfort around the throat and is associated with a high risk of accidental removal of the drainage tube, including self-removal, especially in patients with dementia and disorientation. EGBS is seen to be a more tolerable procedure for patients because of its internal drainage features; however, EGBS does not allow to check the drainage state and has a risk of stent occlusion which can lead to insufficient drainage [17]. In our centers, the ENGBD tube is endoscopically cut inside the stomach for internalization, as conversion from ENGBD to EGBS after improvement of cholecystitis, if necessary. We conducted this study to evaluate the feasibility, efficacy, and safety of endoscopic internalization by cutting an ENGBD tube in the management of acute cholecystitis.

## 2. Materials and Methods

### 2.1. Patient Selection

This retrospective cohort study was conducted at three tertiary care centers: Gifu Prefectural General Medical Center, Gifu University Hospital, and Gifu Municipal Hospital. The database used for this study included clinical data of all endoscopic retrograde cholangiopancreatography (ERCP) procedures performed at these three institutions. The database analysis between January 2010 and December 2019 was then performed to identify patients who met the following inclusion criteria: patients who underwent ENGBD for management of cholecystitis and had attempted endoscopic internalization by cutting the ENGBD tube inside the stomach. However, patients who met the following criteria were excluded: (1) prior history of upper gastrointestinal or biliary tract surgery, except for gastrectomy with Billroth I reconstruction; (2) presence of upper gastrointestinal or pancreatobiliary malignancies; and (3) presence of bile duct stricture, including primary sclerosing cholangitis. All patients provided written informed consent for the endoscopic procedures. The study protocol was approved by the Institutional Review Board of each institution (Gifu Prefectural General Medical Center: 777), and was registered in the University Hospital Medical Network (UMIN) Clinical Trials Registry (UMIN000048263). All procedures performed in this study were in accordance with the ethical standards of the institutional and national research committee and the 1964 Helsinki Declaration and its later amendments or comparable ethical standards.

### 2.2. Procedure

**Figure 1:**
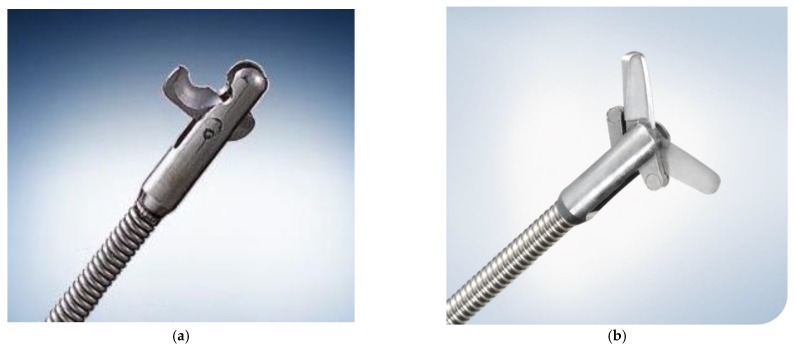
Cutting devices. (**a**) Loop cutter. (**b**) Surgical scissors.

**Figure 2:**
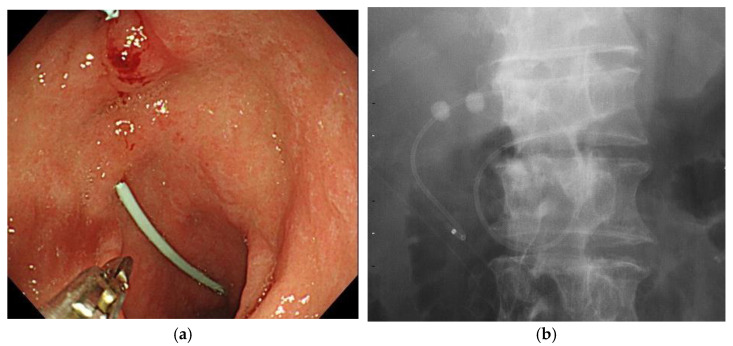
EGBS after internalization by cutting the ENGBD tube. (**a**): Endoscopic image. (**b**): Fluoroscopy image. EGBS, endoscopic gallbladder stenting; ENGBD, endoscopic nasogallbladder drainage.

After successful bile duct cannulation with a duodenoscope (JF260V, TJF260V, or TJF290V; Olympus Medical Systems, Tokyo, Japan), cholangiography was performed to evaluate the shape of the biliary system, including bifurcation of the cystic duct. A 0.025/0.035-inch guidewire (Hydra Jagwire, Boston Scientific, Marlborough, MA, USA; VisiGlide2, Olympus, Tokyo, Japan; or M-Through, ASAHI INTECC Co. Ltd., Aichi, Japan) was advanced into the cystic duct and subsequently into the gallbladder. A hydrophilic guidewire (0.025/0.035-inch NaviPro, Boston Scientific, Marlborough, MA, USA; 0.025/0.035-inch Radifocus, Terumo, Tokyo, Japan) was used to search the cystic duct if advancing the primary guidewire into the gallbladder was difficult. Even in cases where the cholangiogram failed to show cystic duct bifurcation, exploration of the cystic duct by guidewire was performed with or without additional examination using intraductal ultrasonography to recognize the bifurcation of the cystic duct. After successful placement of the guide wire into the gallbladder, endoscopic sphincterotomy (ES) was performed in a standard manner using a papillotome (KD-211Q-0725 or KD-V411 M-0725, Olympus, Tokyo, Japan; Correctome, Boston Scientific, Marlborough, MA, USA) over the guidewire if the patient did not have a history of papillary interventions. A 5-Fr or 6-Fr nasobiliary drainage tube (ENBD) (EN-5S-260P9, Gadelius Medical, Tokyo, Japan; NB-braid, Piolax Medical Device, Kanagawa, Japan) with a pigtail shape at the tip was inserted into the gallbladder as ENGBD. At this time, if aspiration and sufficient rinsing of the gallbladder content is difficult due to bloody pus with high viscosity, cholecystitis was initially managed with ENGBD. After improvement of cholecystitis, esophagogastroduodenoscopy (EGD) was performed. The ENGBD tube was cut using a loop cutter (13B1X00277000029; Olympus, Tokyo, Japan; Figure 1a) in conjunction with surgical scissors (13B1X00277000028; Olympus, Tokyo, Japan; Figure 1b) inside the stomach (Video). After cutting the ENGBD completely, the proximal part of the tube was removed through the nose and the procedure was completed. (Figure 2).

### 2.3. Study Outcomes, Definitions and Statistical Analysis

The primary endpoints of this study were the technical and clinical success of the endoscopic internalization by cutting ENGBD tube. Technical success was defined as successful cutting of the ENGBD tube inside the stomach, and clinical success was defined as the ability to discharge the patient without tube-related complications and recurrence of cholecystitis after internal drainage. The secondary endpoints were the rate of procedural-related adverse events (AEs), procedure time, and the rate of late AE (>7 days). The severity of cholecystitis was evaluated based on the acute cholecystitis severity criteria of the Tokyo Guidelines 2018 [18], and any AEs related to endoscopic procedures were evaluated according to the American Society of Gastrointestinal Endoscopy (ASGE) lexicon [19]. Continuous variables were described as a median and interquartile range (IQR). Comparisons were made using the Fisher exact test or the Pearson’s chi-squared test, as appropriate, for categorical variables and the Mann–Whitney U test for continuous variables. Missing data were covered with mean imputation method. Loss of follow-up was treated as censoring with the last follow-up date. A two-sided *p*-value of < 05 was considered statistically significant. Statistical analyses were performed using JMP version 12 (SAS Institute, Inc., Cary, NC, USA) or R version 3.3.1 (R Foundation for Statistical Computing; http://www.R-project.org/).

## 3. Results

**Figure 3:**
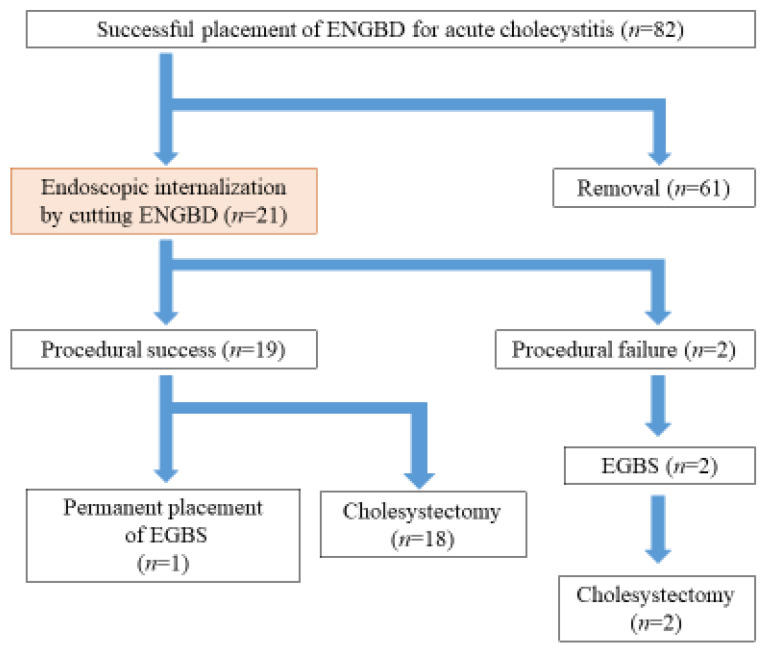
The flow of the patients. ENGBD, endoscopic nasogallbladder drainage; EGBS, endoscopic gallbladder stenting.

A diagram of the patient enrollment is shown in Figure 3. Eighty-two patients underwent ENGBD for acute cholecystitis during the study. The tube was removed in 61 patients after improvement of cholecystitis. As a result, 21 patients who underwent endoscopic internalization by cutting the ENGBD tube were retrospectively examined. Patient characteristics are summarized in Table 1. Imaging studies showed cholecystolithiasis in 21 patients (100%), cystic duct stones in 1 patient (4.7%), and common bile duct stone (CBDS) in four patients (19.0%). The median common bile duct (CBD) diameter was 7.1 mm (range: 4.5–9.4). The severity of cholecystitis was mild in 13 (61.9%) patients, moderate in 7 (33.3%), and severe in 1 (4.7%).

The clinical outcomes are summarized in Table 2. The size of the ENGBD tube was 5-Fr in 20 patients (95.2%), and 6-Fr in one patient (4.7%). The technical success rate of this procedure was 90.5% (19/21) (95% CI, 0.710–0.973) with a clinical success rate of 100%. The median procedural time was 5 min (range: 2–14 min), and the devices used for tube cutting were a combination of loop cutter and surgical scissors in 15 patients (71.4%) and only loop cutter in 6 patients (28.6%). In the two patients who experienced procedural failure, the tip of the ENGBD tube migrated into the CBD from the gallbladder during the procedure. Regarding procedure-related AE, tube migration during the procedure was the only AE, with a rate of 9.5% (2/21) (Table 2). In both patients with failed internalization, ERCP was performed again for EGBS placement, and EGBS was placed until surgery. The median hospital stay was 11 days (range: 6–31), and all patients were discharged within 2 days after the procedure. As for late AE, stent migration was observed in one patient (4.7%), which was confirmed by preoperative abdominal radiographs 31 days after the procedure, but the patient did not have any symptoms of stent migration. As no recurrence of cholecystitis was observed, cholecystectomy was performed as scheduled without EGBS replacement. Among the 19 patients with successful internalization, elective cholecystectomy was performed in 18 (94.7%). Laparoscopic cholecystectomy was performed in all cases, and none of the patients converted to open surgery. The stent was withdrawal by EGD in 7 patients at the day before cholecystectomy. There were no findings of mucosal damage. In 10 patients, the stent was removed during cholecystectomy, and 1 patient, the stent displaced spontaneously. The median waiting time for elective surgery was 61 days (range: 19–103). During the waiting period for elective surgery, no AEs, such as recurrent cholecystitis and cholangitis, were identified.

## 4. Discussion

In this study, endoscopic internalization by cutting the ENGBD tube in the stomach was performed in 21 patients, with a technical success rate of 90.5% (19/21) and a clinical success rate of 100% (19/19). The median procedure time was short, at 5 min (range: 2–14 min). The procedure-related AE was drainage tube migration in 2 patients. In both patients, ERCP was repeated for EGBS placement. Cholecystectomy was performed without recurrence of cholecystitis in 18 of 19 patients who had technical success. The remaining 1 patient was managed with permanent placement of EGBS due to being a poor candidate for cholecystectomy.

**Figure 4:**
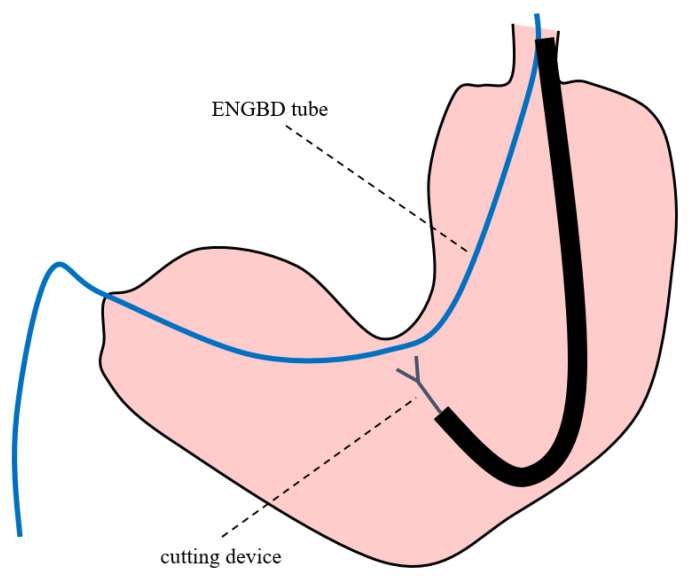
Endoscopic nasogallbladder drainage tube was cut by loop cutter and surgical scissors at the angle of the stomach.

**Figure 5:**
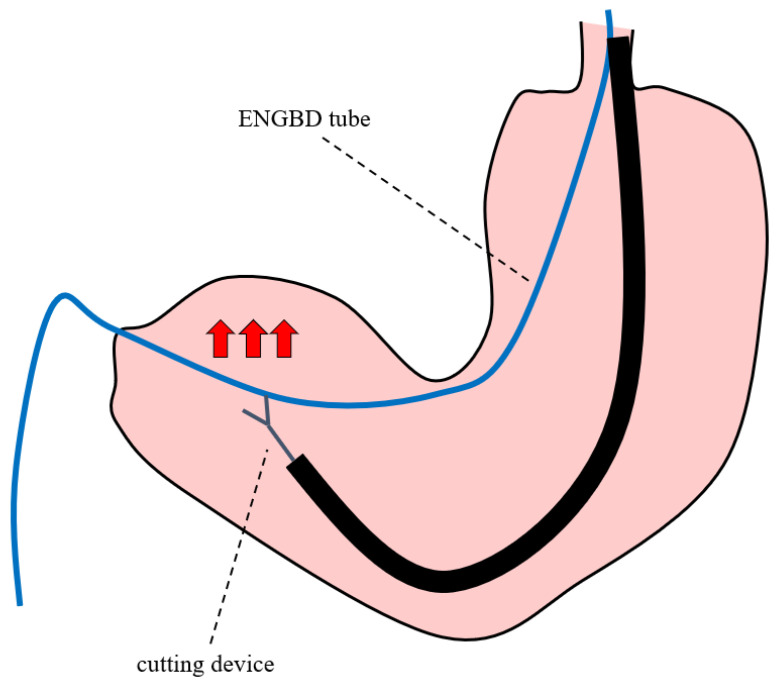
Endoscopic nasogallbladder drainage tube was cut at the antrum of the stomach. Red arrows indicate the direction in which the ENGBD tube is moved during endoscopic cutting.

Although the technical success rate was high, the procedure was unsuccessful in 2 of the 21 patients who underwent attempt internalization. To cut the tube efficiently, it might be better to cut the tube at the angle of the stomach by applying a slight pulling tension to the ENGBD tube, as shown in Figure 4. In this method, the tube can be cut relatively easily because it can be fixed at the angle of the stomach. In addition, when selecting the cutting device, it was difficult to snick the tube with surgical scissors. Therefore, a loop cutter was more efficient in snicking the tube as a starting point, followed by cutting with surgical scissors. Reviewing the causes of the two unsuccessful cases, repeated attempts to cut the tube using a loop cutter at the antrum of the stomach caused the deflection of the tube inside the stomach, which led to migration of the ENGBD tube. The cutting maneuver in the antrum may be challenging because the tube could not be fixed the gastric wall (Figure 5). Regarding the size of the ENGBD tube, 5-Fr catheters were used in 20 patients (95.2%), and 6-Fr were used in one patient (4.7%) in this study. It was still possible to cut the 6-Fr tube with a large diameter, although the procedure time (procedure time of 11 min) tended to be longer than that with 5-Fr catheters (median procedure time of 5 min). Thus, a 5-Fr tube might be better if there is a possibility of internalization by cutting ENGBD.

One of the concerns after endoscopic internalization by cutting the ENGBD tube is recurrence of cholecystitis due to tube obstruction. However, no recurrence of cholecystitis or cholangitis was observed in the median waiting period for cholecystectomy of 61 days (range: 19–103 days) in this study, although EGBS migration was observed in one patient as a late AE. To date, some reports have examined the patency period of EGBS. Kawano et al. [20] retrospectively investigated 18 patients who underwent EGBS for acute cholecystitis and cholecystectomy after improvement of cholecystitis. In this study, a 7-Fr × 15 cm bilateral pig-type stent was used as EGBS, and it was reported that there was no recurrence of cholecystitis with a median waiting period for elective surgery of 72 days (range: 11–118). Doi et al. [17] also evaluated the patency period of EGBS for preoperative drainage. In this study, a 5-Fr or 6-Fr ENBD with a pigtail shape at the tip of the tube was inserted into the gallbladder, followed by aspiration of the gallbladder content and sufficient rinsing with saline until the infected bile disappeared. The ENBD tube was cut to the appropriate length and reinserted into the gallbladder as a stent (EGBS), and the rest of the tube was used as a pusher catheter. It has been reported that in 37 successful cases of EGBS placement, the median waiting period for elective surgery was 42 days (range: 12–346), and no late AEs such as recurrence of cholecystitis and cholangitis were observed. Similar to these reports, the endoscopic internalization by cutting the ENGBD tube that we performed in this study was considered to be a safe procedure for preoperative drainage management of acute cholecystitis. In addition, even if an ENGBD with a small diameter of 5-Fr was selected, cholecystitis recurrence was not observed in the report by Doi et al. [17]. These results suggest that the patency of the tube and straightening of the bile duct allow bile to flow around the tube and prevent cholecystitis recurrence [21]. Based on these results, it is considered appropriate to use a 5-Fr small-diameter tube for the preoperative management of cholecystitis.

Considering the merits and demerits of the two drainage methods of ENGBD and EGBS, a treatment strategy that first manages with ENGBD and then changes to EGBS after improvement of cholecystitis might be useful in cases of cholecystitis with viscous infected content or severe cholecystitis in which the monitoring of proper drainage is desirable. However, the most significant disadvantage of this strategy is that additional ERCP is required to convert ENGBD to EGBS, which increases the risk of AEs and incurs costs for devices and human resources. Therefore, conversion of ENGBD to EGBS by cutting the drainage tube inside the stomach using EGD, as shown in this study, can be a good treatment option because it can be easily performed with a relatively short procedure time and without additional ERCP. However, a comparative study has not yet been conducted to define which preoperative management method between converting from ENGBD to EGBS or removing ENGBD after improvement of cholecystitis is better. A retrospective comparison study was conducted to evaluate the long-term outcomes between permanent EGBS placement (40 patients) and EGBS removal (131 patients) after improvement of cholecystitis in high-risk surgical patients, as previously reported [22]. The cumulative late AE rate was 5.0% in the EGBS group and 22.1% in the removal group (*p* = 0.002), while the recurrence rate of cholecystitis was 5.0% in the EGBS group and 16% in the removal group (*p* = 0.024), with a median follow-up period of 375 and 307 days, respectively. Considering the significantly lower late AE and recurrence rate of cholecystitis, internalization by cutting ENGBD is considered a safer option than simple removal of ENGBD in terms of prevention of recurrent cholecystitis, even with a short period of elective cholecystectomy. Further studies are required to verify this treatment strategy.

This study had limitations. First, the small sample size with a retrospective design at three tertiary centers might cause selection bias and have low external validity. Second, since this study did not have a comparison arm such as a simple removal of the ENGBD tube, further evaluation is required to confirm the efficacy and safety of ENGBD internalization, as described above.

In conclusion, endoscopic internalization by cutting the ENGBD tube after cholecystitis improvement is an effective and safe treatment option and has the potential to prevent recurrent cholecystitis in the waiting period until cholecystectomy. Further comparison studies with larger cohort are required to confirm these findings.

## Figures and Tables

**Table 1:** Patient characteristics.

	*n* = 21
Sex, male, *n* (%)	18 (85.7)
Age, years, median (range)	70 (55–88)
Cholecystolithiasis, *n* (%)	21 (100)
Cystic duct stone, *n* (%)	1 (4.7)
CBDS, *n* (%)	4 (19.0)
CBD diameter, median (range), mm	7.1 (4.5–9.4)
Periampullary diverticulum, *n* (%)	7 (33.3)
Presence of antiplatelet/anticoagulant agents, *n* (%)	6 (28.5)
Previous endoscopic sphincterotomy, *n* (%)	1 (4.7)
Severity of cholecystitis, *n* (%)	
mild	13 (61.9)
moderate	7 (33.3)
severe	1 (4.7)

CBDS, common bile duct stone; CBD, common bile duct.

**Table 2:** Clinical outcomes of the endoscopic internalization by cutting ENGBD.

	*n* = 21
Technical success, *n* (%)	19 (90.5)
Procedural time, median (range), min	5 (2–14)
The size of the ENGBD	
−5 Fr	20
−6 Fr	1
Devices to cut the ENGBD tube, *n* (%)	
loop cutter and surgical scissors	15 (71.4)
loop cutter	6 (28.6)
Procedural-related adverse event, *n* (%)	
migration	2 (9.5)
Hospital stay, median (range), days	11 (6–31)
Clinical success, *n* (%)	19 (100)
Late adverse events (>7 days), *n* (%)	1 (4.7)
migration	1
Recurrence of cholecystitis	0
Cholangitis	0
Elective cholecystectomy, *n* (%)	18 (94.7)
Waiting period for elective surgery, median (range), days	61 (19–103)

ENGBD, endoscopic nasogallbladder drainage; CBDS, common bile duct stone.

## Data Availability

Not applicable.
